# Effectiveness of a training-of-trainers model in a HIV counseling and testing program in the Caribbean Region

**DOI:** 10.1186/1478-4491-7-11

**Published:** 2009-02-17

**Authors:** Cynthia A Hiner, Brinnon Garrett Mandel, Marcia R Weaver, Douglas Bruce, Robert McLaughlin, Jean Anderson

**Affiliations:** 1 Jhpiego, Affiliate of Johns Hopkins University, Baltimore, MD, USA; 2 International Training and Education on HIV [I-TECH], Department of Health Services, University of Washington, Seattle, WA, USA; 3 Adolescent Community Health Research Group, DePaul University, Chicago, IL, USA; 4 Johns Hopkins University School of Medicine, Department of Obstetrics and Gynecology, Baltimore, MD, USA

## Abstract

**Objectives:**

To evaluate the effectiveness and sustainability of a voluntary counseling and testing (VCT) training program based on a training-of-trainers (TOT) model in the Caribbean Region, we gathered data on the percentage of participants trained as VCT providers who were providing VCT services, and those trained as VCT trainers who were conducting VCT training.

**Methods:**

The VCT training program trained 3,489 providers in VCT clinical skills and 167 in VCT training skills within a defined timeframe. An information-monitoring system tracked HIV trainings conducted, along with information about course participants and trainers. Drawing from this database, a telephone survey followed up on program-trained VCT providers; an external evaluation analyzed data on VCT trainers.

**Results:**

Almost 65% of trained VCT providers could be confirmed as currently providing VCT services. This percentage did not decrease significantly with time. Of the VCT trainers, 80% became certified as trainers by teaching at least one course; of these, 66% taught more than one course.

**Conclusion:**

A TOT-based training program is an effective and sustainable method for rapid scale-up of VCT services and training capacity in a large-scale VCT program.

## Background

The United Nations Joint Programme on HIV/AIDS and the World Health Organization estimate that, in low- and middle- income countries, only 10% of individuals who need HIV counseling and testing have access to these services. [1] Identification of HIV infection is the necessary prerequisite and entry point for comprehensive HIV care and treatment. Providing counseling and testing to the growing number of people who need these services calls for an increase in the number of individuals trained to provide them, and on an even broader scale than providers of other HIV services since many more people will require counseling and testing than will go on to require HIV care and treatment services.

The training-of-trainer (TOT) model has the potential to rapidly increase capacity for much needed health services such as HIV counseling and testing by preparing service providers to train other providers in clinical skills. One of the key benefits of this model is that as more trainers are trained, more trainings can be conducted, thus allowing more providers to be trained. This capacity is critical in both achieving rapid roll-out of services and ensuring a continual supply of providers trained to deliver needed services. There will always be some attrition and hence the ongoing need for training of new staff.

The TOT model has been applied in training programs for HIV-related services [2-4] and other clinical areas, [5] but few articles have reported on its effectiveness as it relates to the percentage of participants who actually go on to conduct trainings. Given the potential of the model to rapidly expand capacity, as well as its cost-effectiveness in comparison to traditional training models, [6] this approach will likely find continued use – especially in developing countries dealing with critical public health crises such as HIV/AIDS. Thus, more information is needed about the effectiveness and sustainability of the TOT model in such resource-limited settings, as well as factors that may contribute to its success.

This paper describes the Caribbean Regional Voluntary Counseling and Testing (VCT) Counselor Training Program, which is based on a TOT model. Specifically, we evaluate the program's effectiveness in expanding the VCT service delivery and training workforce based on follow-up data gathered on the percentage of participants trained as VCT providers who were providing VCT services, and the percentage trained as VCT trainers who subsequently conducted VCT skills courses.

## Methods

### VCT Training Program

The Caribbean HIV/AIDS Regional Training (CHART) network and JHPIEGO, an affiliate of Johns Hopkins University, implemented the VCT Training Program in twelve countries within the Caribbean Region to promote regional collaboration, program sustainability, and appropriate distribution of VCT clinical skills and training skills across the region. The TOT model incorporated a combination of competency-based and mastery learning methods applied through a defined "trainer pathway," in which a provider is ultimately able not only to train peers, but also to design and develop curricula for training programs.

#### Training methodology

Competency-based learning is a learning-by-doing training approach that focuses more on correct performance – demonstrating the knowledge, skills and attitudes needed to perform a clinical service according to defined standards – than on simple acquisition of knowledge. Mastery learning also emphasizes correct performance in that participants must demonstrate the competencies associated with the current learning objective before progressing to the next. Together, these approaches help to ensure that participants are able to provide high-quality services upon successful completion of the course.

**Figure 1:**
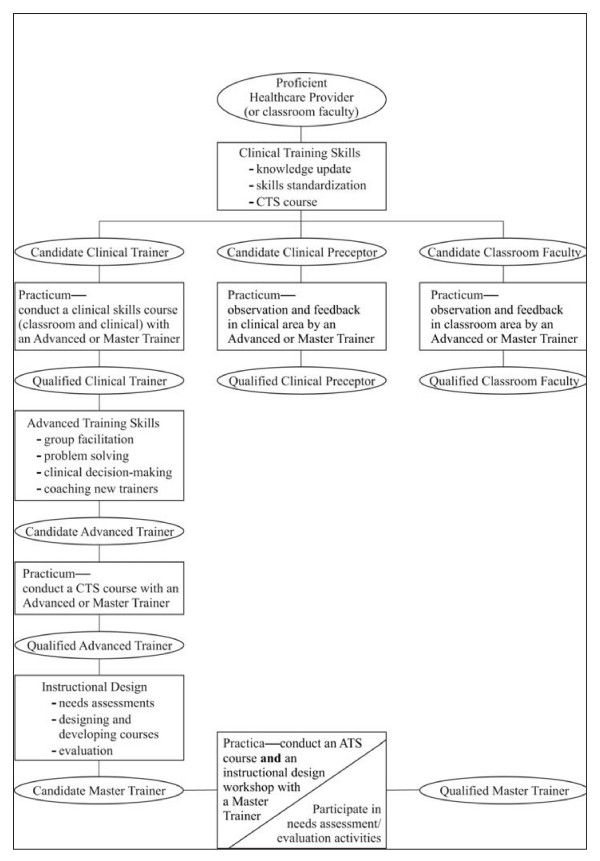
**As this graphic representation of the trainer pathway shows, the process can be applied in building pre-service (faculty in educational institutions) or in-service (trainers in program- or job-based efforts) capacity**. Faculty and trainer development pathway

The trainer pathway is a four-step process that assists clinicians in making the transition from health care provider to clinical trainer, then to advanced trainer and, finally, to master trainer (Figure 1). [7]

▪ First, a health care provider acquires service delivery skills through the clinical skills (CS) course, in this case a course on VCT. To qualify as VCT providers, participants must achieve a minimum of 85% correct responses on a knowledge-based post-test, and demonstrate competency through role plays of various scenarios (e.g., client with positive result, pregnant client with positive result, client with negative result) using a standardized counseling protocol. The course also includes a clinical-based practicum, in which participants practice using the protocol, with supervision, on actual clients.

▪ Once proficient, a provider who has completed the CS course and wants to become a clinical trainer attends a clinical training skills (CTS) course that focuses on how to transfer clinical skills to others. In order to become certified (that is, able to conduct CS courses independently), the prospective trainer must demonstrate competency in conducting one or more CS courses with an advanced or master trainer.

▪ Once proficiency is achieved in conducting CS courses, the clinical trainer who wants to advance to the next level attends an advanced training skills course, which focuses on learning to effectively transfer training expertise to others. The clinical trainer becomes a certified advanced trainer by demonstrating competency in conducting one or more CTS courses with an advanced or master trainer.

▪ Selected advanced trainers may go on to pursue additional training in instructional design to become a master trainer, which is the "top" of the trainer pathway. Master trainers are able to design trainings and conduct advanced training skills courses.

The goal for a training program using this TOT model would be to develop a large number of clinical trainers (who are critical to the rapid expansion of service delivery capacity), a limited number of advanced trainers and even fewer master trainers. The more specialized skills of the latter cadres may not be as urgently needed as those of clinical skills trainers, but are important in ensuring sustainability as the program matures.

#### Participant selection for clinical skills and training courses

The selection of participants for CS courses in each country was based on specific criteria. The suitable candidate would have existing responsibilities related to HIV service delivery; be likely to encounter client populations who would benefit from HIV counseling and testing (including clients accessing antenatal care or treatment for sexually transmitted infections); and demonstrate interest and professional initiative in this program area. Program managers or supervisors deemed to understand the nature and necessity of HIV counseling and testing and be accountable for supporting newly trained VCT providers were also included. Each individual country's ministry of health or national AIDS program was responsible for identifying individuals meeting the above criteria for VCT trainings.

For the selection of participants to attend the CTS course to become trainers, the method varied depending on the country. In larger countries, trainers were selected from each region of the country in order to evenly distribute the training capacity throughout the country and minimize time-off needed (due to travel) to conduct trainings. In the smaller countries of the Organization of Eastern Caribbean States, each country sent four people to a CTS course. Initially, governmental staff – from either the ministry of health or national AIDS program – selected trainees to progress through the training pathway. Later in the project, however, participants with demonstrated proficiency in VCT and interest in becoming trainers were identified by the network of clinical trainers, who then provided feedback to governmental staff to assist in ongoing selections.

### Training Information Monitoring System

The Training Information Monitoring System (TIMS^©^) used in this program is a Microsoft Access database application that tracks and monitors training efforts. For every training event, the system stores information about course content, dates, participants and trainers. For all trainees, it stores information on their qualifications, current place of employment, and contact information, along with courses taken and taught.

In 2004, TIMS was implemented by CHART to track all of the HIV trainings conducted in the Caribbean Region, including those for the VCT program. Data for 2002 and 2003 were entered retrospectively. Information from TIMS was used to generate reports on the number of people trained in clinical skills, clinical training skills and advanced training skills, as well as the number of trainings each clinical trainer and advanced trainer had conducted since the training.

### Follow-Up Activities

Drawing on contact information stored in TIMS, the program team conducted a telephone survey in mid-2005 to follow up on CS course participants whose information had been entered into TIMS through May 2005. Interviewers called sites where participants worked at the time of the CS course. They first asked to talk with the person in charge of VCT services. If he/she was not available, they asked to speak with the CS course participant. For sites in which several people had been trained through the program, inquiries were made in alphabetical order of participant surnames. If neither the person in charge of VCT services nor the participant was available, the interviewer asked to speak with someone familiar with and able to answer questions about VCT services offered at the facility.

In early 2006, an external evaluation of the program was conducted that included analysis of data gathered on CTS course participants through December 2005 – specifically, the percentage who had advanced along the trainer pathway and conducted trainings.

Data analysis was carried out using SPSS and SAS. For those providers no longer at the original site (the site where they were at the time of the training), it was assumed that they were not providing VCT services unless the person interviewed specifically stated that they were. To evaluate the difference in attrition based on time elapsed since training, times elapsed were grouped into three, one-year time periods. Chi-square testing was used to evaluate differences in attrition rates based on the amount of time elapsed since the most recent training. *P *< 0.05 was considered statistically significant.

## Results

**Figure 2:**
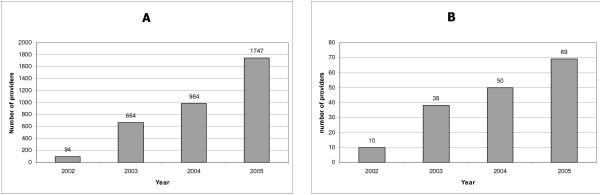
**Total number of VCT providers (A) and VCT trainers (B) trained each year in the Caribbean VCT training program**.

Between June 2002 and December 2005, 3,489 people in the Caribbean Region attended a CS course (Figure 2A) and 167 attended a CTS course (Figure 2B). VCT training activities began in Jamaica in 2002. They were expanded to Trinidad & Tobago, St. Kitts & Nevis, St. Lucia, St. Vincent & the Grenadines, and Surinam in 2003; to Barbados and the Bahamas in 2004; and to Anguilla, Antigua & Barbuda, Dominica, Grenada, and Turks & Caicos in 2005.

### Clinical Skills Course

Data from the telephone survey were analyzed to determine the percentage of CS course participants providing VCT services. For the survey, TIMS data were available for 1,945 people who were trained in VCT from June 2002 through May 2005, whereas data from the subsequent external evaluation showed that 2,432 people had been trained during this time period. This discrepancy is due to a delay in data entry. (TIMS data are manually entered into the system at CHART headquarters in Jamaica after registration forms are mailed in, which causes a delay of several months.) The data from the telephone survey represented trainees proportionately in 11 of the 13 participating countries, while over-representing trainees from Jamaica and under-representing those from the Bahamas.

Of the 1,945 people who were in the system, 55 (3%) provided no information about their current place of employment or contact information, leaving 1,890 people eligible to participate in the study. These 1,890 people worked at 662 unique facilities, and 542 (82%) of these sites participated in the telephone survey, resulting in information on 1,660 people or 85% of those with TIMS data. The sites that did not participate were either unreachable by phone or declined to participate once contacted.

Of the 542 participating sites, 306 were providing both HIV counseling and HIV testing services, 128 were providing counseling or testing only, and 34 were not providing either service. Seventy-four of the sites were places that would not be expected to provide counseling or testing services (e.g., regional offices that did not directly provide health services, insurance offices, restaurants) and were excluded from the analysis. These were the primary places of employment (which is what the TIMS form captures) of individuals who attended VCT training with the intent of providing counseling through a community-based or faith-based organization on weekends or evenings.

Among the 1,660 CS course participants represented in the survey, 1,125 (68%) were currently providing VCT services, as shown in Table 1, and 1,212 (73%) were still working at the original site. Of these, 1,069 (88.2%) were providing VCT services. There were 448 (27%) who were not at the original site, but information obtained confirmed that 56 (12.5%) of them were providing VCT services elsewhere. For most that were no longer at the original site, it was not known whether they were providing VCT services and was assumed that they were not. Of the 1,069 people who were providing VCT services at the original site, information on their current role was available for 1,048. Of these, 560 (53%) provided these services as their primary role and the remainder as their secondary role.

**Table 1:** Follow-up of VCT skills course participants by country

**Country (Total)**	**No. (percentage) at facility and providing VCT**	**No. (percentage) at facility and not providing VCT**	**No. (percentage) not at facility and providing VCT**	**No. (percentage) not at facility and not providing VCT**
Barbados (83)	42 (50.6%)	2 (2.4%)	11 (13.3%)	28 (33.7%)

Jamaica (1,002)	672 (67.1%)	66 (6.6%)	41 (4.1%)	223 (22.3%)

St. Kitts & Nevis (42)	39 (92.9%)	1 (2.4%)	0 (0.0%)	2 (4.8%)

St. Lucia (40)	16 (40.0%)	11 (27.5%)	1 (2.5%)	12 (30.0%)

St. Vincent & the Grenadines (35)	24 (68.6%)	5 (14.3%)	0 (0.0%)	6 (17.1%)

Suriname (85)	64 (75.3%)	6 (7.1%)	1 (1.2%)	14 (16.5%)

The Bahamas (23)	18 (78.3%)	0 (0.0%)	1 (4.3%)	4 (17.4%)

Trinidad & Tobago (350)	194 (55.4%)	52 (14.9%)	1 (0.3%)	103 (29.4%)

**TOTAL (1,660)**	**1,069 (64.4%)**	**143 (8.6%)**	**56 (3.4%)**	**392 (23.6%)**

Whether people were providing VCT at the original site was investigated based on the amount of time that had elapsed since their CS course. There were no significant differences in attrition rates among those trained within the past 1–12 months, 13–24 months, and 25–36 months. Of those who received training in the past year, 67.8% were still working at the same site and providing VCT services compared to 62.8% of those trained 13–24 months ago and 62.1% for those trained 25–36 months ago (Χ^2 ^= 4, *P *= 0.10).

### Clinical Training Skills and Advanced Training Skills Courses

For the second part of the analysis, data available in TIMS at the time of the external evaluation were used to determine the percentage of participants trained as VCT trainers who actually went on to conduct CS courses. A total of 167 people completed the CTS course and, of them, 134 (80%) became certified trainers, as shown in Table 2. The percentage of trainers who were certified varied across countries, from 47% to 100%.

**Table 2:** Number and percentage of VCT trainers and advanced trainers by country

**Country**	**No. qualified as trainers**	**No. certified as trainers**	**Percentage of qualified trainers who were certified**	**No. qualified as advanced trainers**	**No. certified as advanced trainers**	**Percentage of qualified advanced trainers who were certified**
Barbados	11	10	91%	1	1	100%

Jamaica	64	61	95%	16	14	88%

St. Kitts & Nevis	7	6	86%	1	1	100%

St. Lucia	8	7	88%	1	1	100%

St. Vincent & the Grenadines	7	6	86%	1	1	100%

Suriname	10	10	100%	2	0	0%

The Bahamas	13	12	92%	3	3	100%

Trinidad & Tobago	47	22	47%	5	5	100%

**TOTAL**	**167**	**134**	**80%**	**30**	**26**	**87%**

Among the 134 certified trainers, 46 (34%) had taught one CS course, 25 (19%) had taught two courses, 17 (13%) had taught three and the remaining 46 (34%) had taught four or more. Most of the individuals who taught more than four courses were advanced or master trainers. A total of 30 people completed the advanced training skills course and, of them, 26 (87%) were certified as advanced trainers (Table 2). Six of the advanced trainers – five from Jamaica and one from Trinidad & Tobago – subsequently received training in curriculum development and were certified as master trainers.

## Discussion

The VCT training program was effective in developing sustainable VCT service delivery capacity in individual countries within the Caribbean Region, as demonstrated by the fact that almost 65% of CS course participants were confirmed as still providing VCT services. In addition, the program helped build a cadre of trainers who are able to travel and train throughout the region, with a large percentage of participants who had begun the trainer pathway becoming certified as trainers and the majority of those certified (66%) conducting more than one course. The rapid expansion of the program was made possible, at least in part, by the availability of the trainers who were trained through the TOT-based trainer pathway.

The recent evaluation of learning strategies used by United Nations Children's Fund (UNICEF) in resource-limited settings noted that training local professionals to train their colleagues is generally less expensive than sending national or international experts to conduct trainings. [6] In addition, the use of local trainers implementing a TOT model has the advantages of building local capacity as well as ensuring the trainings have cultural relevance and application which will help to enhance learning. Thus, it is likely that the TOT model will continue to be applied in situations where hundreds of training sessions are needed to train thousands of people, and that efforts will be made to mitigate differences in quality through use of competency-based curricula, well-designed training programs and, when needed, implementation of performance and quality improvement methodologies.

### Limitations

The focus of this evaluation was whether people trained in VCT clinical skills were providing these services, and whether those trained in VCT training skills were conducting trainings. It did not address the quality of the services provided or trainings conducted. Although some level of quality is assumed based on the training curriculum and methodologies used, the quality of services should be measured periodically, as feasible. One such related effort has been carried out in Jamaica and found that the quality of services improved through use of a performance and quality improvement process. [8]

The sample for the telephone survey was limited to participants whose data were entered by the end of May 2005, who had provided contact information, and who work at a site that agreed to participate in the survey. Therefore, it is possible that some people who were not contactable or who work at non-participating site are still providing VCT services. In addition, information on the work status of most people who had left the original site was not available, and they were coded as not providing services for the purpose of analysis. It is possible that these individuals, as well, are providing VCT services at another site. All of these factors may have led to an underestimation of the proportion of participants continuing to provide VCT services.

### Findings and Implications

This is the first report on the effect of a TOT training program on the provision of HIV counseling and testing services by trainees. It is important to follow-up on training to see who is on the job and using the skills they have acquired. This information allows a program to determine future training needs, either by site or country. Results on the effectiveness of this TOT model in developing trainers are also significant, providing a basis of comparison for future programs. Our findings are comparable to similar evaluations of TOT models, such as that conducted by UNICEF which found "between 50 and 70% of the TOT trainees going on to provide training to their colleagues." [6]

Although this was a regional program, the lessons learned – in terms of factors contributing to program success and the ways in which challenges were addressed – may be applicable in the implementation of any large-scale training program, such as a national program where training is conducted regionally.

One key factor, which other TOT models have also reported on, in the overall success of this effort was the ongoing support from the different national programs. [3] In this respect, Jamaica's early participation in and adoption of the program were critical because the government recognized the need for distribution of training capacity and was able to harness resources to implement program activities. Following Jamaica's example in successful implementation of the VCT program, regional HIV leaders and program directors recognized the potential efficiencies that could be achieved by scaling up these efforts on a regional level. Throughout the scale-up process, the regional HIV organizations continued to support the collaborative approach by facilitating resources for intra-regional workshops, inter-country travel of master and advanced trainers, and ongoing technical updates for existing trainers to disseminate through their respective training activities. As resources available to the region increased, there was growing awareness among individual countries' governments about the VCT training program. Learning that they could "buy into" the regional capacity without incurring significant costs and contractual obligations (through cost-sharing with other countries), countries were willing to take a collaborative approach to increasing VCT services and training capacity throughout the region.

A strong sense of leadership within the new cadre of VCT advanced and master trainers was another critical factor in the program's success. This encouraged collaboration and accountability among the trainers to travel and expand the program to new countries. Implementation of the trainer pathway, by building local capacity though progressive levels of skill acquisition, may help to cultivate this outcome.

A common challenge in scaling up training activities is that people with demonstrated ability and commitment may not have access to resources to support these activities. Conversely, those who have such access are often too overwhelmed with competing responsibilities. Because the Caribbean is a lower-prevalence setting where there are fewer stand-alone VCT sites, most VCT providers, including those who conduct trainings, have multiple responsibilities – providing antenatal care and treatment for sexually transmitted infections along with VCT services. Such provider-trainers might find it challenging to balance clinical and training roles. However, this is where one of the key benefits of a regional program lies. By enabling participating countries to draw from a collective pool of trainers, the program lessens the burden of individual countries having providers repeatedly take time off from their clinical responsibilities to conduct trainings.

Additionally, the length of the CS course (five days) was sometimes viewed as a barrier for clinicians working at busy practices or for supervisors managing staffing issues. However, since this evaluation was completed, the training curriculum has been modified and is now successfully being implemented in four days. This has resulted in more flexibility for individuals to attend or conduct trainings.

In conclusion, our evaluation of this program demonstrates that a TOT-based regional training program can be successfully implemented for VCT, with the ability to rapidly scale-up human capacity for both service delivery and training in a sustainable fashion.

## Competing interests

The authors declare that they have no competing interests.

## Authors' contributions

CAH conducted the data analysis from the telephone survey and led the writing of the article. BGM contributed to the overall management of the training program and to writing the article. MRW led the analysis of the trainer data, as well as contributed to the literature review and writing the article. DB participated in the analysis of the trainer data, as well as contributed to the literature review and writing the article. RMcL conducted the analysis of the TIMS data for the external evaluation. JA was Principle Investigator for the training program, contributed to writing the article, and critically reviewed and gave final approval of the manuscript for Jhpiego/JHU publication.
